# Erectile Dysfunction and Obstructive Sleep Apnea: A Review

**DOI:** 10.3389/fpsyt.2022.766639

**Published:** 2022-05-26

**Authors:** Yiwei Gu, ChangJing Wu, Feng Qin, Jiuhong Yuan

**Affiliations:** ^1^Andrology Laboratory, West China Hospital, Sichuan University, Chengdu, China; ^2^Department of Urology, West China Hospital, Sichuan University, Chengdu, China

**Keywords:** prevalence, ED, obstructive sleep apnea (OSA), physiopathological mechanism, relationship

## Abstract

Erectile dysfunction (ED) is a disease with a wide scope of etiologies. Obstructive sleep apnea (OSA) is considered one of the risk factors for ED and is less studied. A growing lot of evidence show an association between OSA and ED. This study provides an updated review of the relationship between ED and OSA and the possible physiological mechanisms of ED in patients with OSA based on the current evidence. In clinical interviews, patients with ED may benefit from a sleep evaluation. However, further clinical investigations and more basic research are needed to illustrate the relationship between ED and OSA.

## Introduction

Obstructive sleep apnea (OSA) is characterized by the repeated episodic collapse of the upper airway, leading to arousal from sleep and recurrent oxyhemoglobin desaturation (1). When OSA events occur, the oropharynx at the back of the throat collapses, causing wakefulness, decreased oxygen saturation, or both, which leads to sleep Fragmentation. An estimated 936 million individuals aged 30–69 years worldwide were found to have obstructive sleep apnea; 425 million adults aged 30–69 years have moderate to severe obstructive sleep apnea globally. The number of affected individuals was highest in China, followed by the USA, Brazil, and India (2). The prevalence of OSA in men increases with age, from 10% among those aged 30 to 49 to 17% among those aged 50 to 70, with approximately 24 million persons undiagnosed in the United States (3). OSA has several common sleep and daytime symptoms although the symptoms reported by patients may vary. During sleep, snoring is one of the most collective symptoms and is bothersome to others. The usual symptoms of OSA include inordinate daytime sleepiness or weariness, which reduces the quality of life. Despite acquiring the recommended 7 to 9 h of sleep, feeling unrefreshed is also common in patients with OSA. The severity of OSA is commonly classified according to the apnea-hypopnea index (AHI) value, which is defined as the number of apnea plus hypopnea episodes per hour of sleep. According to expert consensus, a normal AHI is <5 events per hour, 5 to 14.9 is considered mild OSA, 15 to 29.9 is considered moderate OSA, and at least 30 is considered severe OSA (4). A lack of consistency in the definitions of hypopnea influences the AHI value (5).

Erectile dysfunction (ED) has been described as the inability to obtain or maintain a sufficiently firm erection for satisfactory sexual intercourse (6). In the Massachusetts Male Aging Study, the prevalence of ED among men aged 40 to 70 was ~50%, and in the same age group, the prevalence of complete ED was 15% (7). The pooled prevalence of ED was 49.69% in mainland China and increased with age (8). ED is generally considered to be associated with natural aging, other diseases, medical treatment, or changes in emotional state (9).

Regardless of the origin, ED or OSA has a strong negative influence on health and quality of life. In the past, OSA was often overlooked when patients with ED sought treatments. However, the relationship between OSA and ED has been increasingly studied since Guilleminault et al. (10) first reported an underlying association between ED and OSA in 1977. Both disorders are known to be associated with a variety of systemic diseases (e.g., diabetes, hypertension, depression, and other psychological factors) (11). A number of scholars have evaluated the prevalence of ED in patients with OSA (12, 13), and possible mechanisms have been proposed, including an abnormal sympathetic nervous system, endothelial dysfunction, chronic intermittent hypoxia, sleep fragmentation, and/or rapid eye movement (REM) sleep disturbances (14). In this study, we summarized and updated current evidence for OSA and ED and the possible mechanisms underlying the development of ED in patients with OSA, which may help prioritize public health policies and fund public health initiatives and health care planning.

## Materials and Methods

We conducted a thorough literature search based on computerized databases, including PubMed, EMBASE, and SCOPUS, for English-language studies on the association of ED with OSA and related mechanism studies without time restrictions and searched the references of the retrieved papers for additional relevant articles. After the first search, 329 articles were identified. Subsequently, 33 articles were included in this review. The characteristics of the included studies are presented in Tables 1, 2.

**Table 1:** The characteristics of the included clinical studies.

**References**	**Study design**	**Population**	**Mean age (range)**	**ED diagnostic methods**	**OSA diagnostic methods**
Schiavi et al. (15)	Cross-sectional	70	45–75	NPT psychosexual interview	Partial PSG (AHI ≥ 5)
Heruti et al. (16)	Cross-sectional	3,363	36.1 ± 6.8	SHIM Questionnaire	SQQ
Hanak et al. (17)	Cohort	827	64 (51-90)	Brief Male Sexual Function Inventory	Questionnaire (self-reported)
Andersen et al. (18)	Cross-sectional	467	20–80	Questionnaire	PSG
Szymanski et al. (19)	Cross-sectional	90	Non-OSA: 54.5 ± 8.3 OSA: 53.7 ±5.3	IIEF-5	Berlin Questionnaire
Chen et al. (20)	Cross-sectional	60,326	46.4 ± 13.1	Ambulatory claim database	ICD-9-CM PSG
Chen et al. (21)	Cross-sectional	53,335	Non-OSA: 53.8 ± 16.6 OSA: 48.2 ±14.4	ICD-9-CM	ICD-9-CM
Chung et al. (12)	Cross-sectional	6,180	47.8 ± 13	ICD-9-CM	ICD-9-CM PSG
Bozorgmehri et al. (22)	Cohort	2,857	76.2 ± 5.5	IIEF-5	PSG
Pressman et al. (23)	Cross- sectional	31	58.4 ± 6.8	NPT	PSG
Hirshkowitz et al. (24)	Case–control	275	50 ± 9.0– 58 ± 9.8	NPT	PSG
Hirshkowitz et al. (25)	Cross- sectional	1,025	54 (20-82)	NPT	PSG
Chediak et al. (26)	Cross- sectional	37	52.2 ± 14	NPT	PSG
Seftel et al. (27)	Cross- sectional	285	53 ± 13 (16-81)	Medical History	Cleveland Sleep Habits Questionnaire
Fanfulla et al. (28)	Case–control	50	48 ± 11.9	Self-reported Electrophysiologic	PSG
Margel et al. (11)	Cross-sectional	209	AHI <5: 43.95 ± 11.8; AHI > 5 to <20: 44.31 ± 11.8; AHI >20 to <40: 49.88 ± 9.56; AHI >40: 50.19 ± 8.36	IIEF-5	PSG
Teloken et al. (29)	Cross-sectional	50	48 ± 10	IIEF-5	ESS > 10
Shin et al. (30)	Case–control	59	Control: 42.9 ± 9.9; Cases: 44.6 ± 9.8	KIIEF-5	PSG SAQLI ESS
Budweiser et al. (31)	Cross-sectional	401	ED: 61.7 (53.7–69.6); No-ED: 49.9 (42.0–55.4)	IIEF-15	PSG ESS > 10
Stannek et al. (32)	Case–control	186	Control: 46.6 ± 13.7; Cases: 51.1 ± 11.4	Modified IIEF	PSG ESS
Petersen et al. (33)	Case–control	1,493	50.6 ± 10.3 (30-69)	Fugl-Meyer Life Satisfaction Checklist; Brief Sexual Function Inventory	PSG ESS
Gurbuz et al. (34)	Case–control	39	Control: 42.3 ± 7.9; Cases: 41.0 ± 8.8	IIEF-5	PSG ESS
Santos et al. (35)	Cross-sectional	62	52.16	IIEF-5	Polygraphic cardiorespiratory sleep study
Bouloukaki et al. (36)	Cross-sectional	404	42.6 ± 9.3 (18-65)	IIEF-5	PSG ESS
Jeon et al. (37)	Cross-sectional	713	44.8 ± 12.4	KIIEF-5	PSG ESS SAQLI
Popp et al. (38)	Cross-sectional	381	ED: 60.7 ± 11.2; No-ED: 49.0 ± 9.4	IIEF-15	PSG ESS VIGIL-S1
Taken et al. (39)	Case–control	55	43.09 ± 11.48	IIEF-15	PSG
Pascual et al. (40)	Clinical trials	150	ED:48.7(1); No-ED:54.8(1)	IIEF-15	PSG
Schulz et al. (41)	Clinical trials	94	51.5 ± 0.9	IIEF-5	PSG

*PSG, polysomnography; IIEF, The International Index of Erectile Function; ESS, Epworth Sleepiness Scale; SAQLI, Calgary Sleep Apnea Quality of Life Index; VIGIL-S1 test, Vigil-vigilance; KIIEF, Korean version of the IIEF; NPT, nocturnal penile tumescence; SQQ, Sleep Quality Questionnaire; SHIM, Sexual Health Inventory for Men; ICD-9-CM, The International Classification of Diseases, Ninth Revision, Clinical Modification*.

**Table 2:** The characteristics of the included basic science studies.

**References**	**Models**	**Time course**	**Treatments**	**Outcomes**
Soukhova-O'Hare et al. (42)	CIH mice	5 or 24 weeks	Tadalafil	Sexual activity was suppressed after CIH. Decreased expression of endothelial NOS after CIH. Tadalafil treatment significantly improved erectile function.
Liu et al. (43)	LTIH rats	6 weeks	Apocynin	LTIH markedly attenuated the erectile responses, and these effects were partially prevented by apocynin treatment
Zhu et al. (44)	CIH rats	5 weeks	N-acetylcysteine (NAC)	Administration of NAC before CIH significantly improved CIH-induced impaired erectile function
Liang et al. (45)	CIH rats	8 weeks	miR-301a-3p-enriched exosome	miR-301a-3p-enriched exosome treatment significantly recovered erectile function

*CIH, Chronic intermittent hypoxia; LTIH, Long-term intermittent hypoxia*.

### The Relationship Between OSA and ED

A total of nine studies (12, 15–22) evaluated the relationship between OSA and ED in the general population. Three studies (15, 17, 22) reported no statistically significant association between OSA and ED. Szymanski et al. (19) reported a higher prevalence of ED in patients with OSA with a history of myocardial infarction. The severity of OSA was found to be positively correlated with low sexual satisfaction (17). Chen et al. (20) reported an increasing ED incidence in the Chinese OSA population. Another study (21) showed that the incidence of ED was higher in patients with OSA after adjusting for relevant confounders for ED or OSA.

The frequency of OSA in patients with ED was investigated in five studies, with a range of 28–78.92% (23–27). Hirshkowitz et al. (24) found that hypertensive patients with ED had more severe OSA than patients without ED, and abnormal respiratory activity during sleep was found in both hypertensive and normotensive groups with erectile problems. One study (25) showed that the prevalence of OSA in 1,025 patients with ED was 43.8% with an AHI ≥ 5, 27.9% with an AHI ≥ 10, and 19.6% with an AHI ≥ 15, which increased dramatically with increased age in patients with ED. Seftel et al. (27) found that persistent waketime sleepiness or fatigue is not associated with ED.

A total of thirteen studies (11, 28–39) reported ED among sleep clinic populations. The incidence of ED in patients with OSA ranges from 40.9 to 80% (19, 28–31, 34–39). Fanfulla et al. (28) found an association between OSA severity and altered bulbocavernosus reflex. Conflicting results existed regarding the relationship between OSA severity and ED. Furthermore, three studies (11, 32, 36) reported that patients with more severe AHI showed ED with higher severity. Jeon et al. (37) suggested that both a low sleep apnea quality of life index and depressive symptoms were independent risk factors for ED in patients with OSA. Budweiser et al. (31) found that waketime sleepiness was not associated with ED. Popp et al. (38) investigated the association between ED and impaired vigilance performance among patients with OSA and found that the severity of erectile function was significantly associated with vigilance performance regardless of other known common risk factors for ED or OSA.

There are two studies of clinical trials evaluating the association between ED and OSA (40, 41). Pascual et al. (40) conducted a cross-sectional study assessing ED prevalence in patients with OSA and found that the prevalence of ED was 51% in patients with OSA. This study suggested the potential usefulness of ED screening in patients with OSA. In another study, 94 men with severe OSA were prospectively evaluated for the presence of ED. The result showed that 64 of the 94 patients were diagnosed to suffer from ED (41).

Some common shortcomings of quality assessment are evident among these observational studies: inadequate control selection, incomplete and short-term follow-up, and oversight of assessing confounding variables such as smoking, alcohol consumption, and testosterone levels. In addition, ED was diagnosed based on interviews or questionnaires (for instance, the international index of erectile function-5, IIEF-5) in most studies (46). The IIEF-5, which was developed in 1997, is a tool widely used to assess erectile function (6). However, questionnaire-based evaluations are very subjective and can be affected by educational level (47). Some specialized diagnostic tests, including the nocturnal penile tumescence and rigidity (NPTR) test and Doppler ultrasound, are also used to diagnose ED. The NPTR is generally used to distinguish psychogenic ED from organic ED (48). Hence, more prospective studies are needed to elucidate the relationship between ED and OSA.

### Pathophysiological Mechanisms of ED in OSA

Although a high prevalence of ED in the male population with OSA has been detected, no literature reports the precise mechanism for the development of ED in patients with OSA. Penile erection is a neurovascular event modulated by psychological factors and hormonal status. An erection develops after sexual stimulation, which is accompanied by an increase in intracellular cyclic guanosine monophosphate with neurotransmitters and relaxing factors from the cavernous nerve terminal and endothelial cells in the penis, resulting in a several-fold increase in the blood flow in the penis, and penile tunica albuginea occludes venous outflow (49). Therefore, any process that impairs either the neural or vascular pathways that contribute to erection can lead to ED. ED can be classified as psychogenic, organic (neurogenic, hormonal, arterial, cavernosal, or drug-induced), or mixed psychogenic and organic mechanisms. The risks of OSA and ED share some factors, including alcohol abuse, cigarette smoking, and obesity. Some mechanisms have been proposed for ED in OSA, including hormonal, vasculogenic, neurogenic, and psychogenic mechanisms.

The leading theory is that frequent hypoxic events during sleep are an independent risk factor associated with ED (31). A cascade of vascular and inflammatory events is activated by intermittent hypoxic events, including downregulation of circulating nitric oxide (NO) levels, vascular endothelial dysfunction, and the release of oxygen radicals. In the physiology of erection, NO is a crucial transmitter. A decrease in circulating NO levels may also contribute to diminishing nocturnal penile tumescence (50, 51). Endothelin is a potent vasoconstrictor that is stimulated and produced by disturbed endothelial cell regulation and hypoxia and can impair penile tumescence, thus contributing to ED in patients presenting OSA (52).

Neural mechanisms, including peripheral neuropathy and high sympathetic activities, have been considered potential causes of ED in patients with OSA (28, 53). The severity of peripheral nerve axon and myelin injury contributes to impaired erection (54). Fanfulla et al. (28) highlighted peripheral nerve involvement by evaluating the somatosensory evoked potentials of the pudendal nerve and the bulbocavernosus reflex in patients with OSA. Patients with OSA have been reported to have higher sympathetic activity during sleep, especially rapid eye movement sleep (55). The elevated level of circulating norepinephrine not only has an effect on erectile function but also leads to hypertension, which is a risk factor associated with penile arterial insufficiency (56).

Psychological mechanisms have also been proposed. Depression is a common comorbidity in men with ED (57) and may result in ED by reducing sexual desire and/or inhibiting parasympathetic nerve activities (58). Additionally, OSA can cause psychological changes and neural impairment (59). Peppard et al. (60) found a causal link between sleep-related breathing disorder (SRBD) and depression in their long-term investigation. Therefore, individuals with OSA may be at an increased risk of developing psychogenic or mixed psychogenic and organic ED.

Additionally, a hormonal effect of testosterone has been proposed as a potential mechanism for ED with OSA. Testosterone is the primary reproductive hormone in men and is secreted through the stimulation of luteinizing hormone and follicle-stimulating hormone. The hypothalamic-pituitary-testicular axis regulates the amount of testosterone synthesis (61–63). Many studies have found that the serum testosterone level of male patients with OSA is low, and the AHI and oxygen saturation index are negatively correlated with testosterone levels (64, 65). Additionally, low testosterone levels are often accompanied by decreased sleep-related erections, notably below 200 ng/dl (66). Gambineri et al. (67) found lower testosterone levels in obese patients with OSA than in obese control patients and a negative correlation between oxygen saturation and testosterone levels. On the other hand, testosterone plays a role in the pathogenesis of sleep apnea, and supplementation with testosterone may aggravate OSA (68). Also, aberrant reproductive hormone secretion may lead to infertility in male patients. A case-control population-based study found that OSA increases the risk of infertility in male patients, and the risk is associated with the OSA exposure time interval (69). Interestingly, sexual dysfunction occurs frequently in men of reproductive age, causing infertility in some instances where ED and premature ejaculation are common types of male sexual dysfunction, which have a prevalence of one in six infertile men (70). There may be a possible link between ED, OSA, and infertility in men, which needs further research to investigate. Recently, new studies showed that the penis may also be an organ with a circadian rhythm and that disruption of the sleep-wake cycle may impair erectile function (71). Sleep fragmentation may disrupt the diurnal rhythm of testosterone levels (72). Normal NPTR includes 3 to 6 periods with tumescence lasting for 10 to 15 min with a rigidity of at least 70% at the penile tip (73). Nocturnal penile tumescence (NPT) can regulate oxygen-required biological processes to protect the integrity of the corpora cavernosa (74). Although the exact regulatory mechanism of NPT is still unclear, some studies have shown that NPT may be related to neurovascular mechanisms and hormone regulation (75). A diminished or an absence of erections is thought to result from a decrease in REM sleep in patients with OSA (76). As the circadian clock has an effect on physiological functions in many tissues (77), Vignozzi et al. (78) proposed whether a penile clock exists. Nocturnal hypoxia effects should not be ignored when investigating potential pathogenic mechanisms underlying ED and OSA. A study demonstrated reciprocal regulation between hypoxia signaling and the circadian clock, where an intact circadian clock could protect heart cells (79). To date, no core circadian rhythm-related factors have been studied in the penis.

However, limited basic research exists regarding the pathophysiologic basis of ED with OSA. OSA is a multicomponent, heterogeneous disease that can lead to a variety of comorbidities. The intricacies of the disease reduce the possibility of patient investigation, particularly at the tissue level, which limits the understanding of the pathophysiology of ED in OSA and the development of specific treatment. Dr. Gozal's group demonstrated that chronic intermittent hypoxemia was associated with decreased libido and reduced expression of endothelial nitric oxide synthase (NOS; eNOS) in the penis in a murine model (42). They also found no changes in testosterone and neuronal NOS (nNOS) and inducible NOS (iNOS) immunoreactivity after intermittent hypoxia. In another study, Liu et al. (43) demonstrated that long-term intermittent hypoxia (LTIH) was associated with a marked influence on erectile function and was attributed to increased NADPH oxidase activity in a rat model of sleep apnea. The same group in 2015 demonstrated that endoplasmic reticulum stress-related cell apoptosis is involved with low levels of constitutive NOS and NO in rats (44). Recently, Liang et al. (45) showed that exosome treatment significantly improved erectile function in LTIH exposure-induced ED rats and corpus cavernous smooth muscle cells (CCSMCs) by inhibiting apoptosis and promoting autophagy, which were associated with the PTEN/hypoxia-inducible factor-1 alpha (HIF-1α) and Toll-like receptor 4 (TLR4) signaling pathways.

Erection, which is a physiological event involving psychological, neurological, endocrinological, and vascular systems, may be influenced by any reduction in this cooperation. Asking about sleep situations seems rational in clinical interviews for ED, and examinations for ED and gonadal suppression should also be recommended for patients who have severe OSA and should be reevaluated after OSA treatment.

## Conclusion

Overall, the current evidence suggests that OSA may be a risk factor for ED, and basic research on the mechanisms of ED in OSA is limited. However, since only English-language studies were included in this review, we will broaden our search including different languages in the future. Further clinical trials with good designs and more pathophysiological mechanism studies are needed to elucidate the causes and find new definitive therapies.

## Author Contributions

YG and CW designed this study. YG, CW, and FQ took part in the data collection and analyzed the data. YG drafted the manuscript. JY revised the final manuscript. All authors contributed to the article and approved the submitted version.

## Funding

This work was supported by the Natural Science Foundation of China (Nos. 81871147 and 82071639) and the Chengdu Science and Technology Program (2019-YFYF-00087-SN).

## Conflict of Interest

The authors declare that the research was conducted in the absence of any commercial or financial relationships that could be construed as a potential conflict of interest.

## Publisher's Note

All claims expressed in this article are solely those of the authors and do not necessarily represent those of their affiliated organizations, or those of the publisher, the editors and the reviewers. Any product that may be evaluated in this article, or claim that may be made by its manufacturer, is not guaranteed or endorsed by the publisher.
